# Mucormycosis infection associated with global COVID-19 pandemic - an institutional histopathological study

**DOI:** 10.4317/medoral.25130

**Published:** 2023-02-18

**Authors:** R Keerthika, Anjali Narwal, Mala Kamboj, Anju Devi, Rahul Anand, Sivakumar N, Virendra Singh, Varsha Agarwal, Ambika Gupta

**Affiliations:** 1 MDS. Junior resident. Department of Oral and Maxillofacial Pathology and Microbiology, Pt. Bhagwat Dayal Sharma University of Health Sciences, Post Graduate Institute of Dental Sciences (PGIDS), Rohtak, Haryana, India; 2 MDS. Professor. Department of Oral and Maxillofacial Pathology and Microbiology, Pt. Bhagwat Dayal Sharma University of Health Sciences, Post Graduate Institute of Dental Sciences (PGIDS), Rohtak, Haryana, India; 3 MDS. Senior Professor and Head. Department of Oral and Maxillofacial Pathology and Microbiology, Pt. Bhagwat Dayal Sharma University of Health Sciences, Post Graduate Institute of Dental Sciences (PGIDS), Rohtak, Haryana, India; 4 MDS. Associate Professor. Department of Oral and Maxillofacial Pathology and Microbiology, Pt. Bhagwat Dayal Sharma University of Health Sciences, Post Graduate Institute of Dental Sciences (PGIDS), Rohtak, Haryana, India; 5 MDS, Assistant Professor, Department of Oral and Maxillofacial Pathology and Microbiology, D Y Patil Dental College and Hospital, Pune, Maharashtra, India; 6 MDS, Senior Resident, Department of Oral and Maxillofacial Pathology and Microbiology, Faculty of Dental Sciences, King George’s Medical University, Lucknow, Uttar Pradesh, India; 7 MDS. Junior resident. Department of Oral Medicine and Radiology, Pt. Bhagwat Dayal Sharma University of Health Sciences, Post Graduate Institute of Dental Sciences (PGIDS), Rohtak, Haryana, India; 8 MDS. Senior Professor and Head. Department of Oral Medicine and Radiology, Pt. Bhagwat Dayal Sharma University of Health Sciences, Post Graduate Institute of Dental Sciences (PGIDS), Rohtak, Haryana, India

## Abstract

**Background:**

Coronavirus disease 2019 (COVID-19) in the recent times have instilled signs of immunosuppression globally which has further precipitated increasing range of opportunistic infections. Mucormycosis is a distressing opportunistic fungal infection with a high incidence and is the third commonest acute invasive infection following candidiasis and aspergillosis. The aim of the present observational study is to delineate the enigmatic histopathological profile between mucormycosis cases seen prior to pandemic (PPM) and pandemic associated mucormycosis (PAM).

**Material and Methods:**

Tissue archives of 105 histopathologically diagnosed cases of mucormycosis were included and analysed for demographical details and histopathological parameters like fungal load and localization, granuloma formation, necrosis, inflammatory infiltrate and tissue invasion.

**Results:**

0ut of 105 included cases, 11/105 (10.48%) were reported PPM and 94/105 (89.52%) PAM. Among 94 cases of PAM, 51/94 (54%) cases also showed COVID-19 positivity, while 43/94 (46%) did not. Of all the histological variables, increased fungal load and necrosis were observed in PAM relative to PPM cases.

**Conclusions:**

The histopathological variables like fungal load, necrosis, granuloma formation and tissue invasion, could help the clinician in assessing the clinical status at the time of tissue diagnosis and improve the treatment accordingly.

** Key words:**COVID-19, fungal load, granuloma, mucormycosis, necrosis.

## Introduction

Mucormycosis is an acute and deadly fungal illness caused by Mucorales fungal species with a high aggressive potential for contiguous spread and a poor prognosis (1). It might present as varied clinical subtypes among which Rhino-orbital Mucormycosis (ROM) affects the paranasal sinuses often leading to orbital involvement associated with high morbidity and mortality (2). Although clinical and radiological features guide the diagnosis of Mucormycosis, occurrence of ambiguities is not uncommon. Thus, histopathological detection of fungal hyphae is still taken as the gold standard for confirmation of the disease.

The health scenario post 2019 has been dominated globally by a novel strain of severe acute respiratory syndrome coronavirus 2 (SARS-CoV-2) which has multisystemic implications like cardiovascular abnormalities, renal failure, strokes and a high propensity for pulmonary symptoms (3). With a backdrop of failure of respiratory function and immune dysfunction invasive fungal infections like Mucormycosis can spontaneously set in (1).

Mucormycosis tends to occur along with several risk factors including uncontrolled diabetes mellitus (DM), prolonged use of corticosteroids, haematological malignancies, solid organ or hematopoietic stem cell transplantation, human immunodeficiency virus infection, intravenous drug abuse and advanced age which ultimately leads to severe immunosuppression serving as a common ground in COVID-19 patients as well (1,3).

The clear cut association between the drastic rise of Mucormycosis in COVID-19 patients still remains a gray area although several hypotheses have been suggested in the literature. The most widely accepted one is the adjunct of corticosteroids combined with microangiopathy in diabetes and chances of peripheral microthrombi in COVID-19 patients makes the host immunocompromised (affects the ability of macrophages to prevent the germination of the spores of these fungi) and forms the appropriate milieu for development of Mucormycosis (3).

There have been multiple endeavours in reporting the time-sensitive Mucormycosis in COVID-19 inflicted patients but no study has been yet formulated which documents the difference between the histopathological spectrum of Mucormycosis prior pandemic and pandemic associated. Since the global healthcare system is presently burdened with dealing with the secondary complications associated with COVID-19, a better knowledge about the pathogenesis and the histological characteristics of this deadly invasive fungal infection might help in enhancing the treatment modalities.

**Figure 1:**
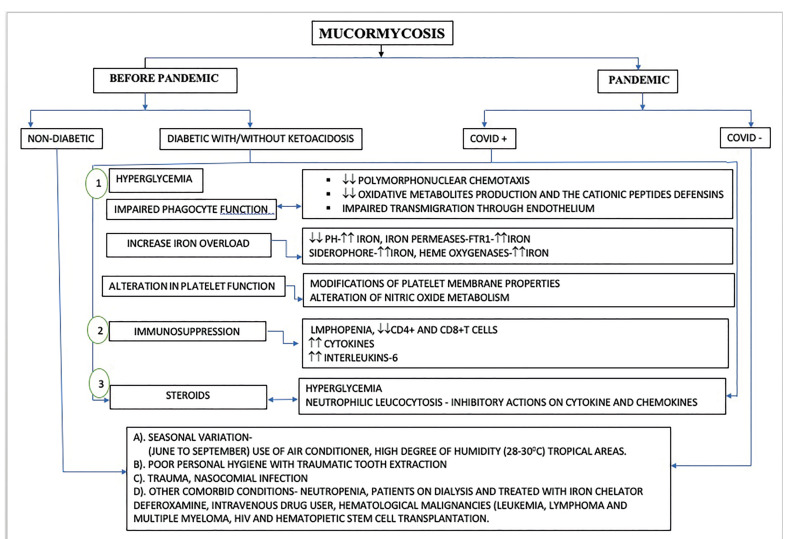
Flowchart showing the pathogenesis of mucormycosis prior and pandemic associated mucormycosis cases.

In this present study we aimed at studying the enigmatic histopathological realm of Mucormycosis in prior pandemic and pandemic associated cases with particular emphasis on fungal load and localization, granuloma formation, necrosis, and tissue invasion in haematoxylin and eosin (H&E) stained tissue sections in the hope that it will shed some light on its progression. An attempt has also been made to hypothesize the possible pathophysiology of Mucormycosis in prior pandemic and pandemic associated patients (Fig. 1).

## Material and Methods

The present observational study with retrospective measure was conducted in the Department of Oral Pathology and Microbiology, PGIDS, Rohtak, Haryana from April to August, 2021 after getting the Institutional Ethical Clearance (PGIDS/BHRC/21/46). 105 histopathologically diagnosed cases of Mucormycosis were collected from archives and split into two groups. Group-I prior pandemic Mucormycosis (PPM) comprised 11 cases of Mucormycosis which reported before COVID-19 pandemic, whereas Group-II pandemic associated Mucormycosis (PAM) consisted of 94 cases that occurred during the pandemic period.

Inclusion criteria:

1. Cases with suggestive clinical features and microscopic confirmation of characteristic broad, pauci-septate or aseptate, ribbon like hyphae with wide-angle branching diagnostic of Mucormycosis were included in study group.

Exclusion criteria:

1. Clinically suspicious cases lacking histopathological corroboration or those treated in outside hospitals were excluded from the study.

Cases during PP and PAM were subcategorized as:

Group 1 - PPM cases with diabetes

Group 2 - PPM cases without diabetes

Group 3 - PAM cases with diabetes, COVID-19 history and other comorbidities

Group 4 - PAM cases with diabetes, COVID-19 history and no other comorbidities

Group 5 - PAM cases with non-diabetic, COVID-19 history and other comorbidities

Group 6 - PAM cases with non-diabetic, COVID-19 history and no other comorbidities

Group 7 - PAM cases with diabetic and non COVID-19 history

Group 8 - PAM cases with non-diabetic and non COVID-19 history

Haematoxylin & Eosin stained tissue sections of all the included cases were scanned at 40x/high power field (HPF) magnification by two independent observers (KR & AN) for histopathological parameters such as fungal load and localization, granulomatous response, necrosis, inflammatory infiltrate, tissue invasion and alteration in maxillary sinus lining.

2. The fungal load was quantitated at several HPFs and graded as occasional (1+), mild (2+), moderate (3+) and marked (4+) respectively (4,5).

3. Similarly, granuloma formation was categorized into absent/occasional (0/1+), mild (2+), moderate (3+) and marked (4+) depending on the quantity of granulomatous foci (4,5).

4. The predominant localization of fungal element at several sites including necrotic areas, granulomas, soft tissue without granuloma, soft tissue & bone, soft tissue necrosis & bone, and solitary bone tissue was recorded. Necrosis were sub-divided into, absent/occasional (0/1+), identifiable (2+), noTable (3+), and extensive (4+) (5).

5. Likewise, inflammatory response was categorized into absent, mild (1+), moderate (2+) & marked (3+). Invasive fungal sinusitis was recorded as present, only when hyphal forms were identified within mucosa, blood vessel or bone, otherwise considered as absent (4).

**Figure 2:**
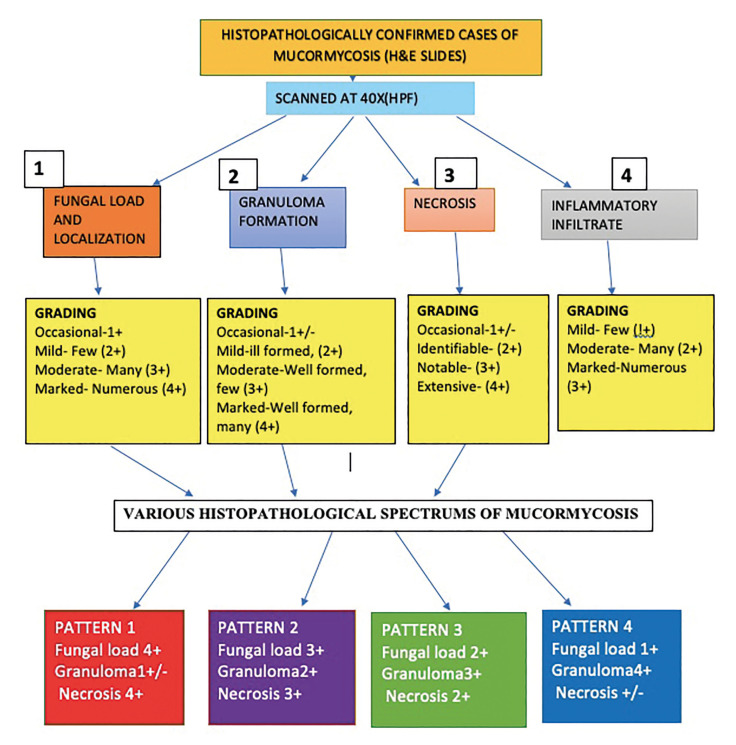
Flowchart showing the algorithm of methodology and evaluation criteria.

Fig. 2 explains the algorithm of methodology and evaluation criteria based on which four patterns (1-4) were categorized delineating increased granuloma formation with increase in grade and simultaneous decrease in fungal load and necrotic areas. This characterization is in accordance with the previous published literature (4,5).

- Statistical test

All statistical analyses were performed using Microsoft Excel worksheet and SPSS Statistics for Windows Version 25.0 (IBM Corp., Armonk, New York, USA). A two-sided *p-value* of less than 0.05 was considered statistically significant in the non-parametric Chi-square test. Association between prior pandemic and pandemic associated Mucormycosis cases with various histological parameters were analysed.

## Results

The present study included 105 cases of mucormycosis, out of which, 11/105 (10%) were reported PPM and 94/105 (90%) PAM cases. Among 94 cases of PAM, 51/94 (54%) cases also showed COVID-19 positivity, while 43/94 (46%) did not. Male predominance was evident with male: female ratio being 2.6:1. The patients age ranged from 2 to 82 years with the mean age of 50.33±14.82 years. Mean age of male patient was calculated to be 49.02±13.79 and female patient is 53.75±16.80. Predisposing factors were identified in 85/105 (79%) patients, among which 73/85 (90%) cases recorded with only DM and 10/85 (10%) with concomitant comorbidities alongside DM. The most common site of involvement was noted in the right maxillary sinus region (31/105-30%) followed by the left maxillary sinus involvement (21/105-20%), bilateral maxillary sinus (13/105-13%), palate (11/105-10%), right maxillary sinus and orbit (11/105-10%), left maxillary sinus and orbit (11/105-10%) and left alveolus (7/105-7%).

- Fungal load

**Figure 3:**
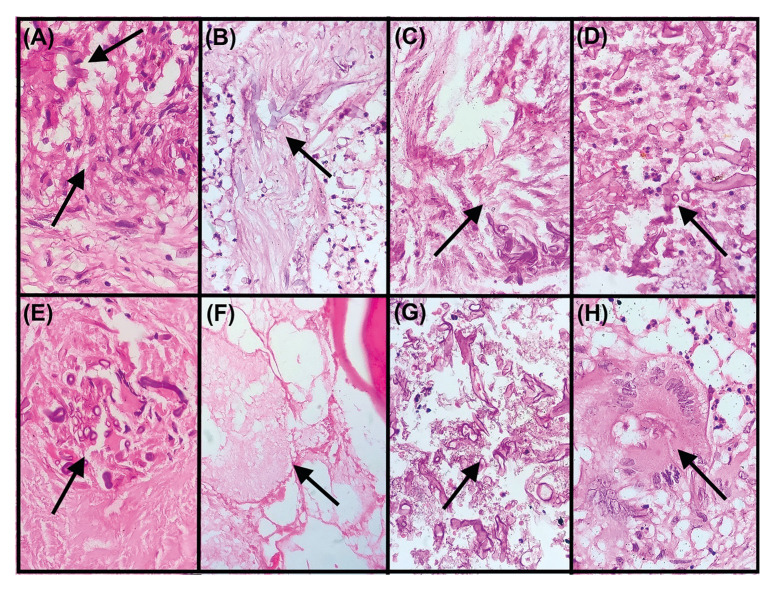
Photomicrograph A-D) depicting the fungal load in mucormycosis cases; A) occasional, B) mild, C) moderate, D) marked (H&E,40x); E-H) illustrating the fungal hyphae localization in mucormycosis cases; E) within granuloma, F) within bone, G) within necrosis, H) within multinucleated giant cell (H&E,40x).

Among PPM cases the occasional, mild, moderate and marked fungal load as shown in Fig. 3 was observed in 0/11(0%), 9/11(82%), 2/11(18%) and 0/11(0%) cases respectively. In cases of PAM it was detected in 0/94(0%), 29/94(31%), 23/94(25%) and 42/94(44%). Comparison of fungal load among PPM and PAM cases was found to be statistically significant (*p* value 0.001) (Table 1).

- Fungal localization

In PPM cases the fungal hyphae localization as shown in Fig. 3 was predominantly found in soft tissue without granuloma (6/11-55%) followed by bone (4/11-36%), soft tissue+ bone (1/11-9%), necrosis (0/11-0%), granuloma (0/11-0%) and soft tissue+ necrosis+ bone (0/11-0%). Among subsequent PAM cases, it was majorly seen in necrotic areas (65/94-69%) followed by granuloma (10/94-12%), bone (6/94-6%), soft tissue without granuloma (6/94-6%), soft tissue+ bone+ necrosis (6/94-6%) and in soft tissue+ bone (1/94-1%). The above comparison was statistically significant with *p* value of 0.002 (Table 1).

- Granuloma

On recording the granulomatous areas in PPM, 7/11 (64%) cases showed absence of granuloma, 0/11 (0%) with mild and 4/11 (36%) with moderate granuloma. In PAM cases, 78/94 (83%) cases were observed with no granuloma formation, 1/92 (1%) with mild and 15/94 (16%) with moderate granulomas. Although there was no statistical significance observed (*p* value 0.242), a larger load of granulomatous foci were observed amongst PPM cases. (Table 1)

- Necrosis

In PPM cases, around 9/11 (82%) cases did not show necrosis whereas 2/11 (18%) cases exhibited identifiable grade of necrosis. In PAM cases, 20/94 (21%) cases presented with no necrosis, while 13/94 (14%) indicated noTable necrosis, 24/94 (26%) with identifiable and 37/94 (39%) with extensive necrosis. Chi-square analysis of the above results was found to be statistically significant (*p* value 0.002) (Table 1).

- Inflammatory infiltrate

Majority of PPM cases were observed with moderate (7/11- 63%) inflammation which was succeeded by mild (1/11-10%) and absent (3/11-27%). A considerable number of PAM cases displayed mild inflammation (41/94-44%) followed by moderate (28/94-30%) and absence (24/94-25%) of inflammation along with a single case of extensive infiltration by inflammatory cells (1/94-1%) (Table 1).

- Tissue invasion

Evaluation of PPM cases presented a single case (10%) with tissue invasion. However, amongst PAM cases, 13/94 (11%) presented with tissue invasion, which was not significant statistically (*p* value 0.662) (Table 1).

**Table 1:**
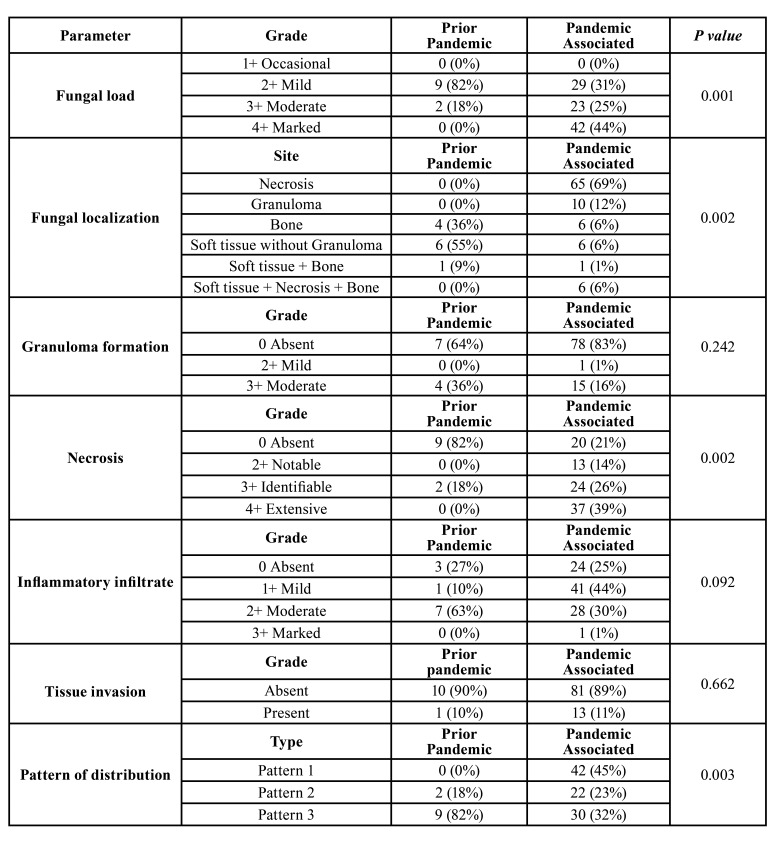
Histopathological variables of PPM and PAM cases.

- Pattern

**Figure 4:**
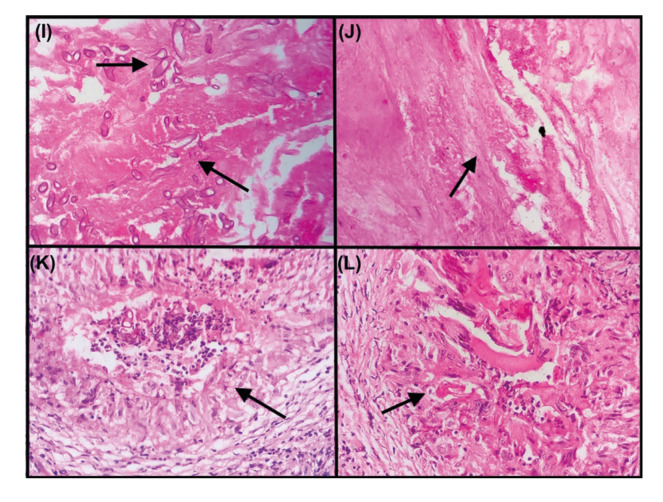
Photomicrograph I-J) illustrating pattern 1 histomorphology comprising; I) marked fungal load (4+) and J) extensive necrosis (4+), without granuloma formation; K-L) illustrating pattern 3 histomorphology comprising; K) mild fungal load-(2+) with simultaneous increase in granuloma formation (3+) and L) identifiable necrosis (2+) (H&E,40x).

Pattern 1 distribution as revealed in Fig. 4 was observed in 42/94 (45%) PAM cases and none of the PPM cases. Pattern 2 was seen in 22/94 (23%) in PAM and 2/11 (18%) PPM cases respectively. Nine (82%) PPM and 30/94 (32%) PAM cases exhibited pattern 3 distribution (Fig. 4), whereas pattern 4 was not observed in any of the study cases. Pattern 3 distribution was majorly seen in PPM cases and pattern 1 was predominantly found in PAM cases as shown in Table 2, which was statistically significant (*p* value 0.003).

**Table 2:**
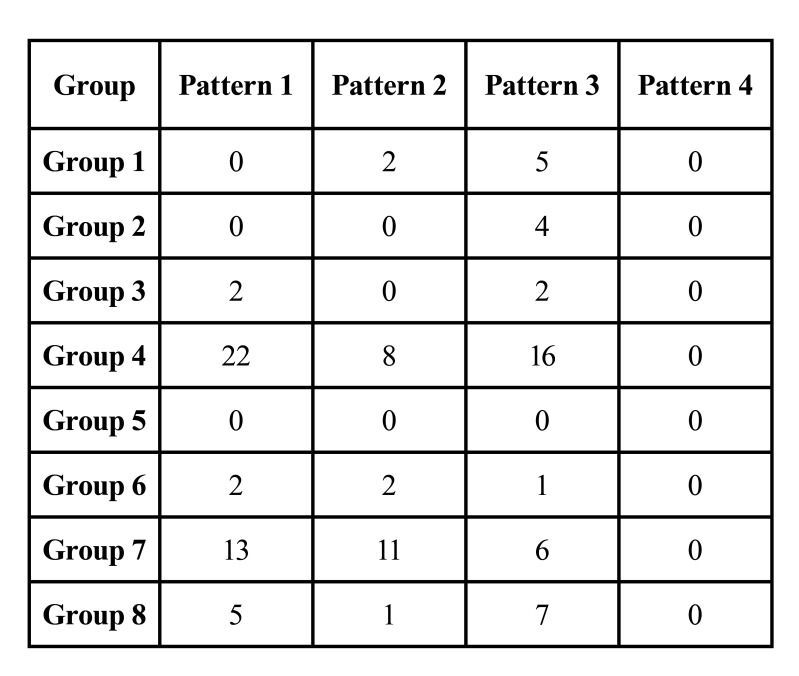
Pattern distribution in various groups of PPM and PAM cases.

## Discussion

ROM is a potentially fatal infection resulting in angioinvasion, mycotic thrombosis and ischemic necrosis of tissues, which could end up in exenteration of the orbit (6). Although mucormycosis has a lower incidence rate varying from 0.005 to 1.7 per million population, a surge in cases have been witnessed recently amounting to a significant increase in its incidence in the wake of the ongoing coronavirus pandemic (7).

In a recent publication, 187 cases of COVID-19 associated Mucormycosis (CAM) in India were reported, with an incidence rate of 0.27% amongst hospitalized cases. Two folds increase in the case load of Mucormycosis has been reported since the last year and currently a staggering 14,872 cases of CAM have been identified in India (8). The extreme humidity along with an annual average temperature range of 28-30°C in the Indian sub-tropical region could be the probable reason for the extensive prevalence of Mucorales as previously discussed in the literature (4).

Our study has an incidence of 49% Mucormycosis among post COVID-19 patients and 41% amongst COVID-19 negative cases amidst the pandemic, while 10% cases were observed prior to pandemic. In a study conducted by Ramaswami *et al*. on 70 post COVID-19 patients diagnosed with Mucormycosis, the mean age was found to be 44.5 years and the disease was more common in males (60%) which is comparable to the present study where the mean age was calculated as 50.33±14.82 years and seen affecting more in males (72%) as compared to females (28%) (9). Roden *et al*. have hypothesised that the effect of oestrogen might be protective in fungal infections which could have led to a lower incidence rate in females (2).

Diabetes Mellitus (DM) is the most frequent co-morbidity in Mucormycosis in about 73.5% in India with the incidence of 1.6 cases/1000 patients. However, in western countries diabetes is associated with only 17% cases of Mucormycosis (10). Diabetes was associated in our study with 98.03% cases of COVID positive Mucormycosis cases. The combination of DM with ketoacidosis and corticosteroid therapy for COVID-19 can synergistically paralyze the function of innate immunity, thereby augmenting the risk of mucormycosis in a susceptible individual (1). With respect to other comorbidities present in our patients, in the present study 10 cases were observed with hypertension, meningioma and renal failure.

The fungal load, explicating the amount of Mucorales in the tissue has an inverse proportionality to the survival rate of the affected individuals was recently reported by Ashina *et al*. who reported a survival of 57% with an increased fungal load (4). We have observed a significantly (*p* value 0.002) higher grade of fungal load in COVID-19 positive cases (46%) subsequent to pandemic rather than the prior pandemic era. It is assumed that the extreme immunosuppression brought about by COVID-19 could favour a higher fungal load which in turn could lead to vast necrotic areas in Mucormycosis cases amid the pandemic.

Granulomas are evolutionarily ancient structures that have been considered as a protective mechanism to destroy or encapsulate foreign materials from further spreading, which elucidate its role in determining the prognosis of Mucorales infections (11). In the present study granuloma formation was observed in 16 PAM cases and 4 PPM cases. Similar studies have investigated the nature and extent of granulomatous inflammation perceived in Mucormycosis and noted moderate to marked granuloma formation in 5 cases which exhibited a relatively higher survival rate (67% to 100%) (4). Additionally, an increased granulomatous response in PPM cases denoted well functional immune system, whereas a decreased granuloma formation noted in PAM cases explicates the failure of the immune system and sub-normal immunity.

Microscopically, we also evaluated varying amounts of necrosis and found vast areas of necrosis in PAM cases (79%), while only a couple of PPM cases (18%) presented with necrosis. In agreement with Ashina *et al*. (4), our observations also disclosed extensive necrosis in some of our cases, and we agree with them in that necrotic sites are the ultimate option to cast about fungal elements. In the present study elevated number of hyphae at the necrotic region in considerable number of COVID-19 positive cases (69%) were observed.

Inflammatory cells play a crucial role in the host defence and we found mild to marked inflammation in PAM cases (70/94 - 75.0%), while it was mild to moderate in majority of Mucormycosis (8/11- 73%) cases which took place during prior pandemic. The data was found to be consistent with the previous study regarding inflammation in Mucormycosis but no consistent association with survival rate and inflammatory infiltration was denoted (4). The COVID-19 infection instils a cytokine storm releasing pro-inflammatory cytokines like IL-6, IL-1, TNF- α and interferon which explains the abundant load of inflammatory cells in COVID-19 positive cases (11).

Mucorales infections are characterized by extensive angioinvasion that contribute to hematogenous dissemination and also thrombus formation which eventually could lead to necrosis (12). We have evaluated our cases for angio, perineural, bone as well as muscular invasion and found tissue invasion in 13 (11%) of PAM cases alongside a single PPM case (7.1%). Ashina *et al*, found angioinvasion in 51% of their study cases and pointed out a decreased survival rate with increased degree of angioinvasion (4). They also recognized a decreased survival rate in patients with increased bone invasion but could not find any instances of perineural invasion in their series. Sravani *et al*. identified consistent perineural invasion in 72% of their samples and recommended it as one of the important histological features indicating extent of invasion in Mucormycosis (13).

While correlating the various patterns in PPM and PAM cases, the preponderance of pattern 3 with granuloma formation was noted in PPM cases (82%), whereas pattern 1 with necrotic predominance was seen in PAM (45%), which could correlate with poorer immuno-compromised status of the patient and dire treatment outcome.

Considering the aforementioned histopathological parameters and their subsequent patterns, these might deduce biological differentiation amidst PPM and PAM and further substantiate as evidence that PAM cases are more aggressive in comparison with PPM cases. However sharing our institutional experience of histopathological spectrum of Mucormycosis reported ahead of pandemic and during pandemic might open a platform for discussion, which could support the clinician in evaluating patient’s progress and thus enhancing better treatment modalities and necessary follow up of such patients. Additionally we attempted to hypothesize the possible pathophysiology of Mucormycosis in prior pandemic and pandemic associated patients as elaborated in Fig. 1.

Our study focused on multiple histopathological facets of Mucormycosis yet the cases does not represent the entire spectrum of this diverse pathology. The follow-up data of the patients were insufficient for proper corroboration.

Using the histopathological variables like fungal load, necrosis,granuloma formation and tissue invasion, our study provides useful insights for clinical and histological profile of COVID-19 positive cases affected with Mucormycosis and which could help the clinicians in assessing the clinical status at the time of tissue diagnosis optimizing the treatment protocol. We endorse a large-scale multi-centric prospective study that would help gain useful data on the main factors aggravating pandemic associated Mucormycosis, thereby managing unprecedented surge of Mucormycosis.
